# Role of Wnt Signaling in Adult Hippocampal Neurogenesis in Health and Disease

**DOI:** 10.3389/fcell.2020.00860

**Published:** 2020-09-16

**Authors:** Sebastian B. Arredondo, Daniela Valenzuela-Bezanilla, Muriel D. Mardones, Lorena Varela-Nallar

**Affiliations:** Institute of Biomedical Sciences, Faculty of Medicine and Faculty of Life Sciences, Universidad Andres Bello, Santiago, Chile

**Keywords:** adult neurogenesis, hippocampus, Wnt, aging, Alzheimer’s disease

## Abstract

Neurogenesis persists during adulthood in the dentate gyrus of the hippocampus. Signals provided by the local hippocampal microenvironment support neural stem cell proliferation, differentiation, and maturation of newborn neurons into functional dentate granule cells, that integrate into the neural circuit and contribute to hippocampal function. Increasing evidence indicates that Wnt signaling regulates multiple aspects of adult hippocampal neurogenesis. Wnt ligands bind to Frizzled receptors and co-receptors to activate the canonical Wnt/β-catenin signaling pathway, or the non-canonical β-catenin-independent signaling cascades Wnt/Ca^2+^ and Wnt/planar cell polarity. Here, we summarize current knowledge on the roles of Wnt signaling components including ligands, receptors/co-receptors and soluble modulators in adult hippocampal neurogenesis. Also, we review the data suggesting distinctive roles for canonical and non-canonical Wnt signaling cascades in regulating different stages of neurogenesis. Finally, we discuss the evidence linking the dysfunction of Wnt signaling to the decline of neurogenesis observed in aging and Alzheimer’s disease.

## Introduction

The subgranular zone (SGZ) of the hippocampal dentate gyrus is one of the neurogenic niches of the adult brain where the generation of new neurons persist during adulthood. Compelling evidence indicate that this process is conserved in mammals including humans (Eriksson et al., 1998; Roy et al., 2000; Coras et al., 2010; Knoth et al., 2010; Spalding et al., 2013; Dennis et al., 2016; Mathews et al., 2017; Boldrini et al., 2018; Moreno-Jimenez et al., 2019; Tobin et al., 2019). New neurons are generated from radial glia-like neural stem cells (NSCs) located in the SGZ, that express nestin, glial fibrillary acidic protein (GFAP) and Sox2 (Kempermann et al., 2004; Bonaguidi et al., 2011). These NSCs, also referred as type 1 cells, are slowly dividing or quiescent, and after activation proliferate asymmetrically and give rise to highly proliferate intermediate progenitor cells or type 2 cells, that transition between type 2a cells and neuronal committed type 2b cells (Kronenberg et al., 2003). Type 2b cells differentiate into neuroblasts or type 3 cells that develop into immature neurons and subsequently to mature granule cells, that become integrated into the hippocampal circuitry (van Praag et al., 2002; Ge et al., 2006; Zhao et al., 2006; Toni and Schinder, 2015). In rodents, these stages are well characterized by morphological features, and the expression of specific markers (Kempermann et al., 2004; Encinas et al., 2011). Among these, doublecortin (DCX) is transiently expressed, from neuronal committed progenitor cells until newborn cells begin to express mature neuronal markers (Brown et al., 2003). Thus, DCX has been a crucial marker used for the identification of newborn neurons in the adult human dentate gyrus (Knoth et al., 2010; Dennis et al., 2016; Boldrini et al., 2018; Moreno-Jimenez et al., 2019; Tobin et al., 2019).

Although the role of adult hippocampal neurogenesis has been challenging to determine in humans, increasing evidence in rodents and non-human primates indicate that adult-born neurons contribute to the structural and functional plasticity of the hippocampus (Snyder et al., 2001; Lacefield et al., 2012; Marin-Burgin et al., 2012; Toni and Schinder, 2015; Drew et al., 2016), and to spatial learning and memory, cognitive flexibility, mood regulation and pattern separation (Deng et al., 2010; Aimone et al., 2011; Denny et al., 2012; Gu et al., 2012; Danielson et al., 2016; Lazarov and Hollands, 2016; Anacker and Hen, 2017), the latter known to be associated to the function of the dentate gyrus in humans (Bakker et al., 2008). Accumulating evidence suggests that dysregulation of adult hippocampal neurogenesis may contribute to cognitive decline in aging and neurological disorders [reviewed in Artegiani and Calegari (2012); Seib and Martin-Villalba (2015); Hollands et al. (2016); Choi and Tanzi (2019)]. Therefore, there has been an evolving interest in the therapeutic potential of strategies aimed to enhance endogenous neurogenesis in conditions affecting cognitive abilities.

Neurogenesis in the adult hippocampus is highly regulated by local environmental cues. The SGZ provides an essential environmental niche for NSCs that allows their proliferation and maintenance, and supports the neurogenesis process (Suh et al., 2009; Schwarz et al., 2012; Faigle and Song, 2013; Toda and Gage, 2018). The neurogenic niche comprises cells, signaling molecules and neurotransmitter components. Growing evidence indicate that Wnt signals are key modulators of different stages of neurogenesis. The first member of the Wnt family was discovered more than 30 years ago (Nusse and Varmus, 1982), and thereafter the interest in Wnts has grown exponentially, since these ligands are involved in diverse developmental and adult processes in health and disease (Logan and Nusse, 2004; Clevers and Nusse, 2012; Jackstadt et al., 2020; Serafino et al., 2020). Wnts are secreted glycoproteins that signal through seven-pass transmembrane Frizzled (FZD) receptors. To date, 19 members of the Wnt family have been identified in mammals, along with 10 members of the FZD family of receptors. Wnt ligands bind to the extracellular cysteine rich domain (CRD) of FZDs to trigger the canonical Wnt/β-catenin signaling pathway (Gordon and Nusse, 2006), or the non-canonical or β-catenin-independent pathways Wnt/planar cell polarity (PCP) (Yang and Mlodzik, 2015; Butler and Wallingford, 2017), and Wnt/Ca^2+^ (Kuhl et al., 2000b; Kohn and Moon, 2005).

Although some Wnts mainly activate one specific Wnt cascade, it also occurs that one Wnt ligand can activate different signaling cascades depending on the receptor and co-receptor context (Mikels and Nusse, 2006; van Amerongen et al., 2008; Grumolato et al., 2010), increasing the possibilities of interaction and the complexity of the Wnt signaling activation. Wnt co-receptors include the single transmembrane low-density lipoprotein receptor-related protein 5 and 6 (LRP5/6) that trigger Wnt/β-catenin signaling activation, the single-pass transmembrane receptor tyrosine kinase-like orphan receptors 1 and 2 (Ror1/2), and Ryk that activate non-canonical Wnt signaling (Bovolenta et al., 2006; Grumolato et al., 2010; Gao et al., 2011; Green et al., 2014). In addition, Wnt signaling is modulated by a number of evolutionary conserved inhibitors and activators [for review see Logan and Nusse (2004); Cruciat and Niehrs (2013)]. Endogenous activators include the family of four secreted glycoproteins R-spondin (RSPO1-4) and Norrin, described as agonists of the canonical Wnt signaling (Cruciat and Niehrs, 2013). Endogenous inhibitors include secreted frizzled-related proteins (sFRPs) composed by five members sFRP1-5, and Wnt inhibitory factor-1 (WIF-1), which directly bind to Wnt proteins preventing their interaction with FZD receptors (Rattner et al., 1997; Hsieh et al., 1999); Dickkopf 1, 2, and 3 (Dkk1-3), which bind LRP5/6 and the transmembrane proteins Kremen to disrupt the interaction of Wnt/FZD (Bafico et al., 2001); and Wise/SOST that bind to LRP5/6 to block Wnt-induced FZD-LRP5/6 interaction (Semenov et al., 2005).

Activation of canonical Wnt/β-catenin signaling involves the formation of Wnt/LRP/FZD ternary complex, which induces the recruitment of the scaffolding protein Disheveled (Dvl), and the multiprotein complex composed of the scaffolding protein Axin, APC, and the enzymes casein kinase 1 (CK1) and glycogen synthase kinase 3β (GSK3-β) (Cong et al., 2004; Zeng et al., 2005; Bilic et al., 2007). In consequence, β-catenin phosphorylation is inhibited, thus preventing its ubiquitination and degradation (Aberle et al., 1997). β-catenin accumulates in the cytoplasm and translocate into the nucleus where it interacts with members of the T cell factor/lymphoid enhancer binding factor (TCF/LEF) family of transcription factors displacing the transcriptional repressor Groucho, and regulating the expression of target genes (Logan and Nusse, 2004; MacDonald et al., 2009). In the Wnt/PCP pathway the binding of the Wnt ligand causes the activation of the small GTPases Rho and Rac, and downstream c-Jun N-terminal kinase (JNK) which regulates cytoskeleton dynamics and activation of activator protein-1 (AP-1) family transcription factors (Jones and Chen, 2007; Yang and Mlodzik, 2015). Other PCP components include the transmembrane proteins Van Gogh-like (Vangl) and Celsr1-3, and the cytoplasmic factors Prickle and Diversin (Jones and Chen, 2007; Yang and Mlodzik, 2015). The Wnt/PCP pathway regulates the coordinated polarization of cells or structures in the plane of a tissue, and orientation of subcellular structures and cellular processes [reviewed in Devenport (2014); Butler and Wallingford (2017)]. The Wnt/Ca^2+^ signaling cascade is a G protein-dependent signaling pathway that triggers the activation of phospholipase C and phosphodiesterase (Kohn and Moon, 2005), increasing the levels of intracellular inositol 1,4,5-triphosphate (IP3) and 1,2 diacylglycerol (DAG) (Koval and Katanaev, 2011). IP3 and DAG lead to the release of calcium from the endoplasmic reticulum and the consequent activation of calcium sensitive proteins such as calcium calmodulin dependent protein kinase II (CamKII) (Kuhl et al., 2000a), protein kinase C (PKC) (Sheldahl et al., 1999) or the phosphatase calcineurin that activates the Nuclear factor of activated T-cells (NFAT) (Saneyoshi et al., 2002; De, 2011).

In the central nervous system, Wnt signaling pathways play pivotal roles during development, controlling cell division, differentiation, polarity, migration, and synaptogenesis (Freese et al., 2010; Bielen and Houart, 2014; Bengoa-Vergniory and Kypta, 2015; Inestrosa and Varela-Nallar, 2015). In the adult brain, Wnt signaling regulates synaptic plasticity, adult neurogenesis and behavior [reviewed in Varela-Nallar and Inestrosa (2013); Oliva et al. (2018)]. Here we summarize evidence supporting that the Wnt signaling is a key regulator of adult hippocampal neurogenesis in health and disease.

## Wnt Signaling in the Regulation of Adult Hippocampal Neurogenesis

**FIGURE 1:**
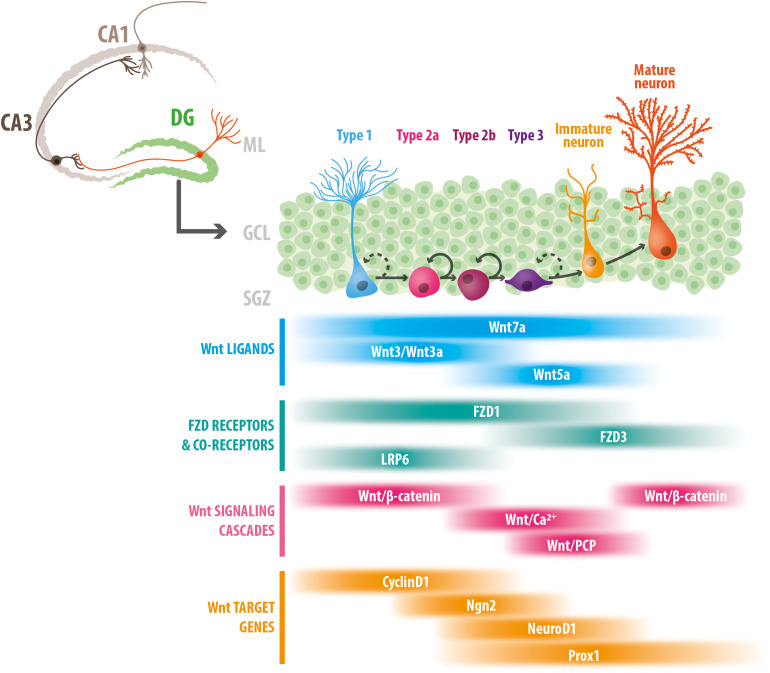
Stage-specific roles of Wnt signaling components in adult hippocampal neurogenesis. Schematic representation of the adult mouse hippocampus, and the stages of neurogenesis in the dentate gyrus. Type 1 NSCs proliferate asymmetrically to give rise to type 2 cells (types 2a and 2b), that differentiate into type 3 cells or neuroblast that develop into immature neurons, and ultimately into mature granule cells. The bottom panel indicates the temporal windows in which Wnt ligands, FZD receptors and co-receptors, Wnt signaling cascades and Wnt target genes have been involved (see text for details). DG, dentate gyrus; ML, molecular layer; GCL, granule cell layer; SGZ, subgranular zone.

Compelling evidence indicate that components of the Wnt signaling pathway play multiple roles during adult neurogenesis. As will be discussed, the data also suggest that canonical and non-canonical Wnt signaling cascades regulate different stages of neurogenesis: Wnt/β-catenin signaling regulates proliferation and fate commitment, while non-canonical Wnt signaling controls the differentiation and development of newborn neurons. In this section, we summarize current knowledge on the role of Wnt signaling components and pathways in controlling different stages of adult hippocampal neurogenesis (Figure 1).

### Wnt Ligands

Wnts are secreted lipid-modified glycoproteins that act as autocrine and paracrine signaling molecules (Rios-Esteves and Resh, 2013; Rios-Esteves et al., 2014). Wnts are expressed in neural progenitor cells (NPCs) isolated from the adult hippocampus (Wexler et al., 2009), and in dentate gyrus astrocytes (Lie et al., 2005; Okamoto et al., 2011). In co-culturing experiments, it was demonstrated that Wnts secreted by astrocytes promote neuronal differentiation of NPCs (Lie et al., 2005; Okamoto et al., 2011). In addition, sequestering Wnts secreted by cultured adult hippocampal progenitors (AHPs) reduced proliferation and the expression of genes involved in the maintenance of progenitors cells, while inducing an upregulation of genes involved in neuronal differentiation (Wexler et al., 2009). This indicates that autocrine Wnt signaling controls maintenance and proliferation of NPCs.

The first study directly linking Wnt proteins and adult hippocampal neurogenesis *in vivo* showed that general blockade of Wnt signaling with a dominant negative of Wnt1 ligand, which non-autonomously blocks Wnt signaling, almost completely eliminated the generation of new neurons in adult rat hippocampus (Lie et al., 2005). On the other hand, lentivirus-mediated overexpression of Wnt3, which is normally expressed in the SGZ and mostly by niche astrocytes (Okamoto et al., 2011), induced neurogenesis in the adult rat dentate gyrus (Lie et al., 2005). In cultured AHPs, overexpression of Wnt3 and Wnt3a, which activate the Wnt/β-catenin pathway (Lie et al., 2005; Kuwabara et al., 2009), increased neuronal fate commitment and enhanced the proliferation of neuroblasts, suggesting that canonical Wnt signaling regulates these processes. In agreement, expression of dominant-negative Lef1 (dnLef1) reduced neuronal differentiation induced by co-culture with hippocampal astrocytes (Lie et al., 2005). Wnt7a was also described as an endogenous modulator of hippocampal neurogenesis that regulates proliferation and neuronal differentiation. Wnt7a knockout mice showed fewer NPCs, which exhibited lengthened cell cycles and a reduced cell cycle reentry, and also showed impaired neuronal differentiation (Qu et al., 2013). On the contrary, chronic infusion of Wnt7a directly into the rat hippocampus increased the number of immature neurons (Ortiz-Matamoros and Arias, 2019). Wnt7a knockdown in NSCs reduced the expression of Cyclin D1, while when NSCs were induced to differentiate into neurons Wnt7a knockdown reduced mRNA levels of neurogenin 2 (Ngn2). In cultured progenitors β-catenin binds to TCF/LEF binding sites in the promoter region of Cyclin D1, while in neurons β-catenin binds to TCF/LEF binding site in the promoter region of Ngn2. These findings indicate that Wnt7a regulates proliferation and differentiation through the canonical Wnt/β-catenin signaling pathway (Qu et al., 2013). In addition, immature neurons in Wnt7a knockout mice exhibited reduced dendritic arborization (Qu et al., 2013). These data indicate that Wnt7a has multiple roles during adult hippocampal neurogenesis controlling proliferation, differentiation and development of newborn neurons.

More recently, Wnt5a was also identified as an endogenous niche factor that regulates hippocampal neurogenesis. We determined that reducing the levels of Wnt5a in the dentate gyrus of adult mice decreased the generation of new neurons (Arredondo et al., 2020). Lentivirus-mediated knockdown of Wnt5a reduced the differentiation of neuronal committed progenitor cells, which remained as non-proliferative intermediate Sox2-expressing progenitors that failed to continue with the neuronal differentiation program. In addition, impaired dendritic arborization of newborn neurons was observed when knocking down Wnt5a. A similar effect was observed when Wnt5a was reduced in cultured AHPs, in which neuronal differentiation and morphological development of the derived neurons were reduced, while treatment with Wnt5a had the opposite effect (Arredondo et al., 2020). In agreement, chronic infusion of Wnt5a ligand into the adult rat hippocampus increased the number of immature neurons and altered their pattern of neurite outgrowth (Ortiz-Matamoros and Arias, 2019). In cultured AHPs, Wnt5a activated CamKII, PKC and JNK (Arredondo et al., 2020), and activated AP1 and c-jun in differentiated but not proliferative AHPs (Schafer et al., 2015), indicating that Wnt5a triggers activation of non-canonical Wnt signaling cascades. Moreover, we found that the effect of Wnt5a on neuronal differentiation was mediated Wnt/Ca^2+^/CamKII signaling, while the effect on morphological development involved Wnt/Ca^2+^ and Wnt/JNK cascades (Arredondo et al., 2020), indicating that Wnt5a is an endogenous factor regulating neurogenesis through non-canonical Wnt signaling.

### Wnt Receptors and Co-receptors

Frizzleds are the primary receptors for Wnt signals. All FZD isoforms present conserved structural characteristics, including a N-terminus extracellular region containing the highly conserved CRD, seven transmembrane regions, and an intracellular C-terminus that mediate the interaction between FZD and Dvl [reviewed in Huang and Klein (2004); Schulte (2010)]. Several FZD receptors are expressed in cultured AHPs, and some of them show specific expression patterns during differentiation (Wexler et al., 2009; Cui et al., 2011; Schafer et al., 2015; Mardones et al., 2016). In the adult dentate gyrus, FZD3 is expressed in immature and mature neurons, but not in NSCs or NPCs, suggesting FZD3 is required for later stages of adult neurogenesis (Schafer et al., 2015). In agreement, FZD3 expression increased upon differentiation in cultured AHPs (Schafer et al., 2015). Retrovirus-mediated knockdown of FZD3 did not affect neuronal differentiation of newborn cells, however, the dendritic arborization of FZD3-deficient newborn neurons was reduced. In addition, the orientation and positioning of these neurons within the granule cell layer (GCL) was affected (Schafer et al., 2015). FZD3 knockdown reduced the Wnt5a-dependent activation of c-Jun and JNK in differentiated AHPs, indicating FZD3 activates Wnt/PCP signaling in these cells. The same study demonstrated that *in vivo* knockdown of Celsr 1-3 impaired the development and maturation of adult-born neurons without affecting neuronal differentiation (Schafer et al., 2015). Celsr1-3, the mammalian homologs of Drosophila Flamingo, are a family of atypical cadherins that contain seven transmembrane segments, and are part of the so-called Wnt/PCP core proteins in vertebrates (Yang and Mlodzik, 2015). Newborn neurons deficient in Celsr 2/3 showed impaired dendritic arborization and altered positioning within the GCL, while Celsr 1-deficient neurons displayed abnormal orientation (Schafer et al., 2015). Celsr 3 knockdown also altered dendritic pruning of adult-born neurons (Goncalves et al., 2016). Altogether, these data suggest that Wnt/PCP signaling is involved in polarization and dendritic development of adult-born neurons, but not in fate commitment.

FZD1 receptor is also expressed in the adult dentate gyrus, where it was found in NSC, NPCs and immature neurons, and its expression is reduced in mature neurons (Mardones et al., 2016), suggesting the role of this receptor is restricted to early stages of adult neurogenesis. We determined that retrovirus-mediated knockdown of FZD1 in the dentate gyrus of adult mice reduced neuronal differentiation of newborn cells, while increasing the differentiation into astrocytes (Mardones et al., 2016). Additionally, FZD1-deficient immature neurons showed altered migration within the GCL, but exhibit normal dendritic arborization (Mardones et al., 2016). FZD1 has been largely described as a receptor for the canonical Wnt signaling, and in agreement, FZD1 knockdown reduced β-catenin levels and the expression of proneural Wnt target genes in AHPs (Mardones et al., 2016). These results suggest that FZD1 regulates neuronal fate commitment through the canonical Wnt/β-catenin signaling pathway. In accordance, knockdown of the co-receptor for the canonical Wnt pathway LRP6, lead to a reduction in neuronal differentiation of newborn cells (Schafer et al., 2015). Interestingly, as observed by FZD1 knockdown, no effect on morphological development was observed in LRP6-deficient newborn neurons. In agreement with its role in early stages of neurogenesis LRP6 is expressed in proliferating AHPs and its expression was reduced upon differentiation (Schafer et al., 2015). Altogether, these evidences suggest that specific receptors and co-receptors activate canonical Wnt/β-catenin to control neuronal fate commitment. Interestingly, β-catenin reporter mouse lines showed a peak of Wnt/β-catenin activity during early stages of adult hippocampal neurogenesis. Different transgenic reporter mouse lines have been used to evaluate the activity of Wnt/β-catenin signaling in the dentate gyrus: the BATGAL mice (Lie et al., 2005; Garbe and Ring, 2012; Heppt et al., 2020), the ins-topGal mice (Garbe and Ring, 2012), and the Axin2^LacZ/+^ mice (Heppt et al., 2020). Although the expression pattern of the reporter activity is not exactly the same in the different mouse lines likely for the molecular construct of the transgenes, the use of BrdU birth-dating strategies and specific molecular markers together with the reporter activity showed that Wnt/β-catenin signaling is active during early stages of adult hippocampus neurogenesis (including NPCs and proliferating neuroblasts), and is attenuated in immature neurons (Lie et al., 2005; Garbe and Ring, 2012; Heppt et al., 2020). Considering that activation of a specific Wnt signaling pathway may antagonize the activation of other Wnt signaling cascades (Ishitani et al., 2003; Topol et al., 2003; Mikels and Nusse, 2006; Grumolato et al., 2010; Sato et al., 2010; Mentink et al., 2018), it is feasible to suggest that the Wnt/β-catenin pathway might be inhibited after fate commitment by the activation of non-canonical Wnt signaling cascades, which as discussed, regulate the development of newborn neurons (Schafer et al., 2015; Arredondo et al., 2020). Interestingly, Wnt/β-catenin activity is reactivated in mature newborn neurons (Garbe and Ring, 2012; Heppt et al., 2020), suggesting that the canonical Wnt pathway might also control later stages of neurogenesis such as maturation or synaptic integration. Notably, it was recently shown that the attenuation of Wnt/β-catenin signaling in early stages of newborn neurons is required for correct dendrite development, and Wnt/β-catenin reactivation in maturing neurons modulates the tempo of dendritic growth and spine formation (Heppt et al., 2020), indicating that a precise control of Wnt signaling activity is required for the generation of new granule cells in the adult hippocampus.

Interestingly, a dual role in adult hippocampal neurogenesis was determined for ATP6AP2 (Schafer et al., 2015), an adaptor protein between Wnt/β-catenin and Wnt/PCP signaling that possess a dual function forming a signalosome to initiate canonical Wnt signaling, and acting as a Wnt/PCP core protein (Buechling et al., 2010; Hermle et al., 2013). ATP6AP2 knockdown in proliferating progenitors reduced the activity of the TCF/LEF in response to Wnt3a, while in differentiated progenitors ATP6AP2 knockdown reduced AP-1 signaling in response to Wnt5a (Schafer et al., 2015). This evidence indicates that ATP6AP2 modulates the activation of canonical Wnt/β-catenin and non-canonical Wnt/PCP signaling in NPCs at different stages of the neurogenic process. *In vivo*, ATP6AP2 knockdown had a dual effect reducing the number of immature neurons and inducing defects in several aspects of the morphological development, migration and orientation of new neurons in the adult hippocampus (Schafer et al., 2015).

Altogether, the discussed evidence suggests that Wnt signaling components mediate the activation of specific signaling cascades, which coordinately control the progression of neurogenesis in the adult hippocampus.

### Soluble Modulators of the Wnt Signaling Pathway

The endogenous Wnt antagonists sFRP3 and Dkk1 have shown to regulate neurogenesis in the adult hippocampus (Jang et al., 2013; Seib et al., 2013). sFRP3 is highly expressed in the dentate gyrus by mature granule cells in the GCL and regulates different stages of neurogenesis (Jang et al., 2013). sFRP3 knockout mice exhibited increased proliferation of NSC, together with increased dendritic development, spine density and accelerated maturation of newborn neurons. Interestingly, in the adult hippocampus there is a septo-temporal gradient of expression of this Wnt inhibitor that is inversely related to NSCs proliferation, suggesting that sFRP3 levels, and therefore Wnt signaling activity, contribute to the graded distribution of neurogenesis in the adult dentate gyrus (Sun et al., 2015). sFRP3 is also involved in the physiological modulation of neurogenesis by electroconvulsive stimulation (ECS) and wheel running, which concomitantly with the increase in neuronal activity, lead to a reduction in the levels of sFRP3 in the dentate gyrus and to the activation of the Wnt/β-catenin signaling pathway (Jang et al., 2013). Besides, Dkk1 regulates self-renewal of NPCs and morphological maturation of newborn neurons (Seib et al., 2013). In NPCs loss of Dkk1 increased Wnt/β-catenin signaling reporter activity, indicating that Dkk1 negatively regulates the canonical Wnt pathway in the adult hippocampus (Seib et al., 2013). Dkk1 was involved in the age-dependent decrease in neurogenesis, which will be discussed later.

### Wnt/β-Catenin Target Genes

Wnt/β-catenin target genes have been involved in multiple stages of adult hippocampal neurogenesis. Cyclin D1 is involved in the Wnt-mediated induction of proliferation in neural progenitors (Shtutman et al., 1999; Tetsu and McCormick, 1999). In proliferative NPCs, β-catenin is bound to the TCF/LEF motif in the Cyclin D1 promoter, associated with the active chromatin markers acetylated histone H3 (AcH3) and trimethylated histone H3 at lysine 4 (H3K4me3). But when NPCs are induced to differentiate, β-catenin dissociate from the Cyclin D1 promoter (Qu et al., 2013). On the contrary, upon differentiation β-catenin binds a TCF/LEF binding site in the Ngn2 gene promoter in association with active chromatin markers AcH3 and H3K4me3 (Qu et al., 2013). Ngn2 is a proneural basic helix-loop-helix (bHLH) transcription factor that promotes neuronal differentiation (Israsena et al., 2004). Prior to differentiation, no β-catenin was detected in the TCF/LEF binding site of the Ngn2 promoter in NPCs.

NeuroD1 is also a bHLH proneural transcription factor involved in the Wnt-mediated induction of neuronal differentiation (Kuwabara et al., 2009). NeuroD1 is expressed in neuronal committed progenitors and immature neurons, but not in NSCs (Gao et al., 2009). Overexpression of NeuroD1 in cultured adult NSC increased their neuronal differentiation, while reducing their differentiation into oligodendrocytes and astrocytes (Hsieh et al., 2004), indicating NeuroD1 promotes neuronal fate-commitment. In the adult dentate gyrus, β-catenin knockdown in Sox2 cells induced the loss of NeuroD1 progenitors, as well as a decrease in newborn granule neurons, with no effect on the NSC pool (Kuwabara et al., 2009). *Neurod1* gene promoter contains a TCF/LEF binding site that is overlapped with a binding site for Sox2 (Sox/LEF site). In undifferentiated NSCs, Sox2 and the histone deacetylase HDAC1 repressor protein are associated with the Sox/LEF site in the *Neurod1* promoter. In differentiated neurons β-catenin, along with acetylated histone H3 and methylated histone H3 at lysine 4, which are related with transcriptional activation, are present in the *Neurod1* promoter (Kuwabara et al., 2009). The data indicate that the *Neurod1* promoter is repressed by Sox2 in NSCs, and in response to Wnt stimulation it is transcriptionally activated by β-catenin leading to NeuroD1 expression and neurogenesis (Kuwabara et al., 2009). The gene encoding prospero-related homeodomain transcription factor 1 (Prox1) also contains TCF/LEF sites overlapped with Sox2 binding sites in promoter/enhancer regions (Karalay et al., 2011). Prox1 is expressed in type 2 cells, neuroblasts, immature neurons and mature granule neurons restricted to the dentate gyrus (Kempermann et al., 2004; Lavado et al., 2010; Karalay et al., 2011). Prox1 is required for the maintenance of intermediate progenitor cells (Lavado et al., 2010), and for neuronal differentiation of granule cells (Karalay et al., 2011). Altogether, these data suggest that in adult hippocampal neurogenesis Wnt/β-catenin signaling regulates proliferation through the expression of Cyclin D1 and promotes neuronal differentiation through the expression of proneural transcription factors including Ngn2, NeuroD1 and Prox1.

## Wnt Signaling in the Decline of Neurogenesis in the Aging Hippocampus

An age-related decline in adult hippocampal neurogenesis has been evidenced in rodents, non-human primates and humans (Kuhn et al., 1996; Gould et al., 1999; Leuner et al., 2007; Olariu et al., 2007; Ben Abdallah et al., 2010; Knoth et al., 2010; Kohler et al., 2011; Dennis et al., 2016; Mathews et al., 2017; Boldrini et al., 2018; Sorrells et al., 2018), suggesting that conserved mechanisms may underlie the reduced capacity of the aged hippocampus to generate new neurons. Recently, a correlation between the loss of immature neurons and an early cognitive decline was determined in aged humans (Tobin et al., 2019), suggesting that efforts to promote neurogenesis may foster new therapeutic possibilities for the aging brain.

**FIGURE 2:**
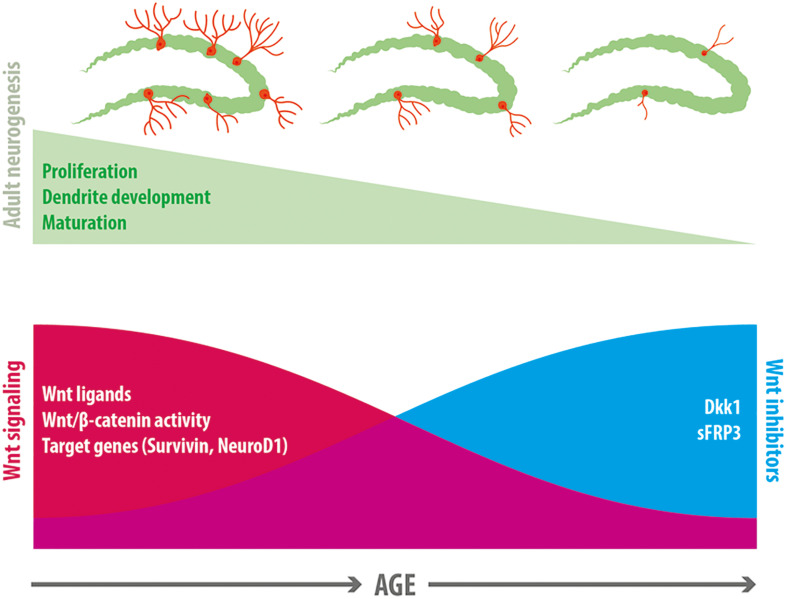
Wnt signaling in the age-related decline in neurogenesis. A reduction in neurogenesis is observed in the dentate gyrus with age, which is accompanied by a decline in proliferation of neural precursor cells, a decreased dendritic development and delayed maturation of adult-born neurons. Evidence exists indicating that a decline in Wnt signaling is associated with this reduction of neurogenesis. In normal aging there is a decrease in the expression of most Wnt ligands in hippocampal astrocytes, a decrease in canonical Wnt signaling activity in the dentate gyrus, and a reduction in the expression of Wnt target genes that control neurogenesis (including Survivin and NeuroD1). Concomitantly, there is an increase in the expression of the Wnt inhibitors sFRP3 and Dkk1 in the hippocampus with age.

The reduced neurogenesis is likely a consequence of a deteriorated neurogenic niche unable to sustain neurogenesis (Hattiangady and Shetty, 2008; Kalamakis et al., 2019). Growing evidence suggest that the Wnt signaling pathway is part of the signaling mechanisms affected, that might contribute to the decline in neurogenesis (Figure 2). In support of this idea, β-catenin reporter mice exhibit a strong decrease in β-catenin signaling activity in the GCL with age, and increasing β-catenin activity counteracts the age-associated maturation defects of adult-born dentate granule neurons (Heppt et al., 2020). In addition, the expression of Wnt3 and Wnt3a in the dentate gyrus decreases with age, concomitantly with the decrease in newborn neurons positive for NeuroD1 (Okamoto et al., 2011). In aged rats (22-month-old) almost no expression of Wnt3 was observed in astrocytes of the SGZ compared to young rats (4-week-old), although the number of astrocytes remained unaffected (Okamoto et al., 2011). This was also determined in cultured primary astrocytes from the hippocampus of aged mice (9-month-old), which showed reduced levels of Wnt3 and Wnt3a compared to astrocytes cultured from young animals (4-week-old). Interestingly, the same study determined that NSCs isolated from the hippocampus of young and aged mice exhibited a more effective neuronal differentiation when cultured on young versus aged primary astrocyte layer. This effect was not observed when Wnt3 was knocked down in young astrocytes (Okamoto et al., 2011), suggesting that loss of Wnt signals might contribute to the impaired neurogenesis in the aged hippocampus. Of note, the expression of FZD receptors and co-receptors were almost unchanged between young and aged NSC (Okamoto et al., 2011). Another study determined that conditioned media from young astrocytes induced promoter activity of the anti-apoptotic protein Survivin in aged and young NPCs, while conditioned medium from aged astrocytes decreased Survivin promoter activity and NPC proliferation compared to control medium. Survivin is a Wnt target gene (Tapia et al., 2006), and lentivirus-mediated expression of Survivin in the dentate gyrus of aged mice (13-month-old), increased proliferation (Miranda et al., 2012). This study also determined that Wnts released by astrocytes promote NPC proliferation by inducing Survivin expression, and that most Wnt ligands are downregulated in aged astrocytes. Interestingly, wheel running, a well characterized inducer of neurogenesis in young and aged hippocampus (van Praag et al., 1999, 2005), induced an increase in the number of Wnt3 expressing cells concomitantly with an increase in the density of immature neurons in the dentate gyrus (Okamoto et al., 2011), suggesting that Wnt3 could mediate the stimulation of neurogenesis in the adult hippocampus.

In addition to the downregulation of Wnt signals, in aging there is an increase in endogenous Wnt inhibitors. Increased levels of Dkk1 were observed in the hippocampus of aged mice (Seib et al., 2013; Kase et al., 2019). Interestingly, loss of Dkk1 restored neurogenesis in old mice (2-year-old) and increased the dendritic complexity of newborn neurons. Moreover, loss of Dkk1 restored spatial working memory and memory consolidation, and improved affective behavior in aged mice (Seib et al., 2013). sFRP3 was also increased in the aging hippocampus (Kase et al., 2019). Interestingly, genetic inhibition of sFRP3 in a mouse model of accelerated aging, rescued neural progenitor proliferation in the hippocampal dentate gyrus (Cho et al., 2019).

## Wnt Signaling in the Impairment of Neurogenesis in Alzheimer’s Disease: Therapeutic Implications

Impaired neurogenesis is observed in several neuropsychiatric and neurodegenerative diseases such as mood disorders, epilepsy, Parkinson’s disease and Alzheimer’s disease (AD) (Lucassen et al., 2010; Winner and Winkler, 2015; Galan et al., 2017; Toda et al., 2019). AD is the most common type of dementia, it is estimated that 30 million people suffer form AD worldwide. AD is characterized by a progressive memory loss, impaired cognitive functions, neuronal loss and synaptic dysfunction. Histopathological hallmarks of AD are the extracellular deposition of amyloid β peptide (Aβ) forming amyloid plaques, and the presence of intracellular neurofibrillary tangles mainly composed by hyperphosphorylated tau proteins [reviewed in Selkoe and Hardy (2016)]. Aβ is generated from sequential proteolysis of amyloid precursor protein (APP) by β- and γ-secretase enzymes (O’Brien and Wong, 2011). In addition to neuronal loss, reduced neurogenesis was evidenced in the dentate gyrus of patients with AD pathology (Li et al., 2008; Crews et al., 2010; Ekonomou et al., 2015; Moreno-Jimenez et al., 2019; Tobin et al., 2019). Post-mortem brain analysis from AD patients revealed a progressive decline in the number of newborn neurons, and in the maturation of these cells as the disease advanced (Moreno-Jimenez et al., 2019). Reduced neurogenesis has also been evidenced in different mouse models of AD, which show impairments in NPCs proliferation, differentiation and maturation of newborn neurons (Donovan et al., 2006; Rodriguez et al., 2008; Demars et al., 2010; Fiorentini et al., 2010; Hamilton et al., 2010; Abbott et al., 2013; Zeng et al., 2016; Choi et al., 2018). Interestingly, in AD mice deficits in neurogenesis precede Aβ plaque and NFT formation, suggesting that impairment in neurogenesis may mediate early cognitive decline (Demars et al., 2010; Fiorentini et al., 2010; Hamilton et al., 2010; Zeng et al., 2016). Recently, reduced number of neuroblasts in early stages of cognitive decline was determined in humans, suggesting that reduced neurogenesis may promote cognitive deficits in AD, or exacerbate them (Tobin et al., 2019). Because increased neurogenesis in the dentate gyrus is associated with improved cognitive capacities (Toda et al., 2019), there has been great interest in the potential of neurogenesis as a therapeutic target for conditions affecting cognition. In this regard, genetic manipulation of neurogenesis by inducing the expression of the proneural gene NeuroD1 in hippocampal progenitors restored spatial memory in a mouse model of AD (Richetin et al., 2015). This evidence supports the potential of neurogenesis as a therapeutic target to prevent or improve cognitive deficits in normal aging and pathological conditions.

Interestingly, we and others have determined that hippocampal neurogenesis is stimulated in AD mouse models through physiological (Hu et al., 2010; Rodriguez et al., 2011; Varela-Nallar et al., 2014; Tapia-Rojas et al., 2016; Choi et al., 2018), and pharmacological stimulation (Fiorentini et al., 2010; Abbott et al., 2013; Varela-Nallar et al., 2015; Choi et al., 2018; Zeng et al., 2019). These evidences demonstrate that NSCs in the hippocampus retains the ability to generate new neurons. In this context, the decrease in neurogenesis in AD could be due to a deterioration of the neurogenic niche. Wnt signaling is likely affected in the SGZ niche since compelling evidence indicate a downregulation of this signaling pathway is associated to the pathophysiology of AD [reviewed in De Ferrari and Inestrosa (2000); De Ferrari et al. (2014); Inestrosa and Varela-Nallar (2014); Oliva et al. (2018)]. Among the several components of the Wnt pathway that are altered in AD, increased levels of Dkk1 were found in post-mortem brains of AD patients (Caricasole et al., 2004), and in the hippocampus of the TgCRND8 mouse model of AD (Rosi et al., 2010), expressing a double mutant form of the human APP. Also, increased levels of active GSK-3β was observed in the dentate gyrus of TgCRND8 mice, suggesting a downregulation of Wnt signaling activity in this area (Rosi et al., 2010). Moreover, in AD patients altered gene expression was found for the soluble Wnt inhibitor WIF-1 in the temporal lobe (Humphries et al., 2015), Wnt7b and intracellular components of canonical Wnt signaling in the entorhinal cortex and hippocampus (Riise et al., 2015), and FZD3 in prefrontal cortex (Folke et al., 2019). In addition, a genetic variant of the Wnt co-receptor LRP6, showing reduced activation of the canonical Wnt signaling has been associated to late-onset AD (De Ferrari et al., 2007; Alarcon et al., 2013).

**FIGURE 3:**
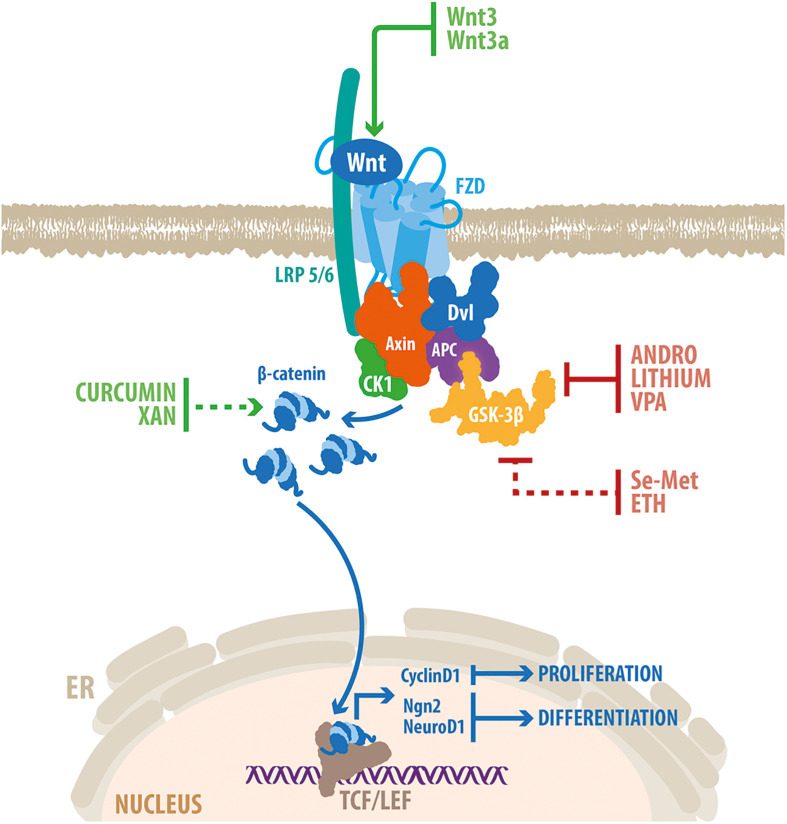
Genetic and pharmacological activation of Wnt/β-catenin promotes neurogenesis in the hippocampus of AD models. Schematic representation of the Wnt/β-catenin signaling pathway. Wnt ligand binds to FZD and LRP5/6, which trigger the recruitment of a multiprotein complex composed also of Axin, APC, CK1 and GSK-3β. This prevents the phosphorylation and degradation of β-catenin that translocates into the nucleus where it binds to members of the TCF/LEF families of transcription factors, to modulate the transcription of target genes. The Wnt/β-catenin signaling components that are target of genetic activation (Wnt3 and Wnt3a) and drugs able to stimulate neurogenesis in the hippocampus of animal models of AD are indicated. Red lines indicate inhibition; green lines indicate activation. Dotted red line indicates GSK-3β inactivation through the PI3K/Akt pathway; dotted green line indicates that the precise mechanism of activation of the Wnt/ β-catenin signaling remains elusive. Some of the drugs (see text for details) have shown to induce the expression of target genes involved in Wnt-mediated induction of proliferation (Cyclin D1) and differentiation (Ngn2 and NeuroD1) in adult hippocampal neurogenesis. VPA, valproic acid; Se-Met, Selenomethionine; ETH, Ethosuximide; XAN, Xanthoceraside.

Considering the crucial role of the Wnt signaling in the regulation of neurogenesis, it might be possible that the dysregulation of this signaling pathway may contribute to neurogenesis deficits observed in AD. Of note, overexpression of Wnt3 restored neurogenesis in the hippocampus of the 5xFAD mouse model of AD (Choi et al., 2018), that express human APP and PSEN1 with a total of five AD-linked mutations. As well, overexpression of Wnt3a was also able to restore neurogenesis levels in the dentate gyrus of 3xTgAD mice, bearing human APP, tau and PSEN1 with AD-linked mutations (Shruster and Offen, 2014). These evidences indicate that in AD brain, neurogenesis is able to respond to exogenous Wnt stimulation, and suggest that Wnt manipulation is an attractive therapeutic target to promote neurogenesis in this pathological condition (Figure 3).

Supporting the association between Wnt signaling impairment and reduced neurogenesis, several drugs able to enhance neurogenesis in AD models have shown to modulate components of the Wnt signaling pathway (Figure 3). Pharmacological inhibition of the key component of the Wnt signaling pathway GSK-3β, enhances neurogenesis in the hippocampus of AD mice (Fiorentini et al., 2010; Varela-Nallar et al., 2015; Zeng et al., 2019). Lithium, a widely used mood stabilizer that inhibits GSK-3α/β by competing with the cofactor magnesium, induced the proliferation and survival rate of NPCs in the SGZ of TgCRND8 mice (Fiorentini et al., 2010). Lithium treatment induced an increase in the number of immature neurons expressing nuclear β-catenin, supporting the activation of Wnt/β-catenin signaling in newborn neurons. Importantly, therapeutic concentrations of lithium induced proliferation of cultured AHPs, which was prevented by β-catenin knockdown (Wexler et al., 2008), indicating that lithium induced neurogenesis trough activation of the Wnt/β-catenin signaling pathway. Additionally, Andrographolide (ANDRO) one of the main constituents of the medicinal plant *Andrographis paniculata* (Panossian et al., 2000; Cheung et al., 2001), that inhibits GSK-3β through a substrate-competitive mode of action (Tapia-Rojas et al., 2015), promoted hippocampal neurogenesis in the APPswe/PSEN1ΔE9 mouse model of AD (Varela-Nallar et al., 2015). ANDRO treatment induced proliferation and increased the density of immature neurons in the dentate gyrus of AD mice, concomitantly with an increase in hippocampal levels of β-catenin and NeuroD1 in the hippocampus (Varela-Nallar et al., 2015). Importantly, ANDRO was shown to improve cognitive performance in APPswe/PSEN1ΔE9 mice (Serrano et al., 2014), and in J20 mice expressing human APP with two mutations linked to familial AD (Cisternas et al., 2019). More recently, Valproic acid (VPA), another selective inhibitor of GSK-3β used as an antiepileptic and mood-stabilizing drug, was shown to promote proliferation, increase the density of immature neurons, and improved learning and memory in the dentate gyrus of triple transgenic APPswe/PSEN1ΔE9/Nestin-GFP mice (Zeng et al., 2019). VPA treatment increased β-catenin levels, and induced the expression of NeuroD1, suggesting the activation of the Wnt signaling pathway in the hippocampus of AD mice.

In addition, the biological trace element Selenomethionine (Se-Met) and Ethosuximide (ETH), which inactivate GSK-3β through the PI3K/Akt pathway, also promoted neurogenesis in AD models (Tiwari et al., 2015; Zheng et al., 2017). Together with the inactivation of GSK-3β, Se-Met increased β-catenin levels, induced the expression of Cyclin D1, and increased cell proliferation and neurogenesis in the hippocampus of a 3xTg AD mice (Zheng et al., 2017). On the other hand, treatment with the antiepileptic drug ETH, reversed cognitive dysfunction, and increased proliferation and neuronal differentiation in the dentate gyrus of a rat model of AD induced by the injection of Aβ (1-42) into the hippocampus (Tiwari et al., 2015). ETH prevented the Aβ-induced reduction in the expression of neurogenesis-related genes (including Ngn2 and NeuroD1), and Wnt signaling components, suggesting that the effects of ETH may be mediated by β-catenin signaling (Tiwari et al., 2015).

Curcumin, a natural polyphenol compound derived from turmeric (*Curcuma longa*), was also suggested to induced neurogenesis through the activation of Wnt/β-catenin signaling pathway. Curcumin encapsulated in PLGA nanoparticles induced NSC proliferation and neuronal differentiation in the hippocampus of an Aβ-induced rat model of AD, and reduced the cognitive deficits (Tiwari et al., 2014). Curcumin enhanced nuclear translocation of β-catenin, decreased GSK-3β levels, and increased promoter activity of Cyclin D1. In the hippocampus, curcumin enhanced the expression of Wnt3a, Dvl, FZD1 and LRP5/6, and the Wnt target genes Ngn2 and NeuroD1, and reduced the expression of the negative regulators of Wnt signaling WIF-1 and Dkk1. Interestingly, pharmacological and genetic inhibition of the Wnt pathway blocked the stimulation of neurogenesis mediated by curcumin, indicating that the effects of curcumin are mediated by activation of Wnt/β-catenin signaling. Another natural product, Xanthoceraside (XAN), a triterpenoid saponin monomer extracted from the husks of *Xanthoceras sorbifolia Bunge*, ameliorated the cognitive impairment and concomitantly increased NSCs proliferation and neuronal differentiation in APPswe/PS1ΔE9 mice (Zhu et al., 2018). Interestingly, XAN treatment enhanced the expression of Wnt3a, increased the levels of inactive GSK-3β and induced nuclear translocation of β-catenin in the hippocampus of APP/PS1 mice, suggesting that XAN may promote neurogenesis by enhancing the Wnt/β-catenin signaling pathway (Zhu et al., 2018). Moreover, Dkk1 inhibited the effects of XAN in cultured NSC.

## Concluding Remarks

The reviewed studies indicate that the Wnt signaling plays multiple roles in adult hippocampal neurogenesis including NPCs proliferation, fate-commitment, development and maturation of newborn neurons. Evidences suggest a stage-specific expression of particular receptors that might activate different Wnt signaling cascades to control the progression of neurogenesis. Although the role of the canonical Wnt co-receptor LRP6 support this notion, the role of other co-receptors that control the activation of non-canonical Wnt signaling remains to be elucidated. The identification of Wnt co-receptors involved in adult neurogenesis is a critical issue that should be addressed to gain a more comprehensive understanding of how canonical and non-canonical Wnt signaling are regulated during adult neurogenesis. In addition, it will be interesting to further study the downstream signaling components and effectors involved in the regulation of adult hippocampal neurogenesis by non-canonical Wnt signaling.

Several studies indicate that Wnt proteins released by hippocampal astrocytes and progenitor cells are crucial components of the SGZ niche. In addition, endogenous Wnt inhibitors are also components of the neurogenic microenvironment that dynamically regulate Wnt-mediated neurogenesis under physiological conditions. Considering the increasing number of Wnt regulators identified to date, it will be interesting to further investigate the contribution of these molecules to the dynamic control of neurogenesis.

In agreement with the critical roles of Wnt signaling in adult neurogenesis, evidence indicates that Wnt signaling is associated with the age-dependent decline in neurogenesis. Concomitantly with the decrease in the generation of new neurons, in normal aging there is a reduction in the expression of Wnt proteins, an increase in the expression of Wnt inhibitors, and a decrease in canonical Wnt signaling activity in the dentate gyrus. Wnt dysfunction might also underlie the impairment of neurogenesis observed in AD. Interestingly, genetic and pharmacological activation of Wnt signaling was shown to restore adult hippocampal neurogenesis, and also to improve cognitive performance in animal models of AD. Although it is not yet known how neurogenesis contribute to hippocampal function in humans, compelling evidence in animal models suggest that adult-born neurons are important for learning and memory, cognitive flexibility and mood regulation. In addition, recent findings support that neurogenesis impairment contributes to cognitive decline in aging and AD. Therefore, a better understanding on the molecular mechanisms involved in the regulation of neurogenesis may have important therapeutic implications. The reviewed evidence suggests that stimulation of Wnt signaling emerges as an attractive strategy to enhance endogenous neurogenesis and improve hippocampal-dependent cognitive function.

## Author Contributions

SBA, DV-B, and MDM wrote and revised the manuscript. LV-N, wrote, drafted, and edited the manuscript. All authors approved the final version as submitted.

## Conflict of Interest

The authors declare that the research was conducted in the absence of any commercial or financial relationships that could be construed as a potential conflict of interest.

## References

[B1] Abbott A. C. Calderon Toledo C. Aranguiz F. C. Inestrosa N. C. Varela-Nallar L. (2013). Tetrahydrohyperforin increases adult hippocampal neurogenesis in wild-type and APPswe/PS1DeltaE9 mice. *J. Alzheimers Dis.* 34 873–885. 10.3233/jad-121714 23302657

[B2] Aberle H. Bauer A. Stappert J. Kispert A. Kemler R. (1997). beta-catenin is a target for the ubiquitin-proteasome pathway. *EMBO J.* 16 3797–3804. 10.1093/emboj/16.13.3797 9233789 PMC1170003

[B3] Aimone J. B. Deng W. Gage F. H. (2011). Resolving new memories: a critical look at the dentate gyrus, adult neurogenesis, and pattern separation. *Neuron* 70 589–596. 10.1016/j.neuron.2011.05.010 21609818 PMC3240575

[B4] Alarcon M. A. Medina M. A. Hu Q. Avila M. E. Bustos B. I. Perez-Palma E. (2013). A novel functional low-density lipoprotein receptor-related protein 6 gene alternative splice variant is associated with Alzheimer’s disease. *Neurobiol. Aging* 34 e1709–e1718. 10.1016/j.neurobiolaging.2012.11.004 23218566

[B5] Anacker C. Hen R. (2017). Adult hippocampal neurogenesis and cognitive flexibility - linking memory and mood. *Nat. Rev. Neurosci.* 18 335–346. 10.1038/nrn.2017.45 28469276 PMC6261347

[B6] Arredondo S. B. Guerrero F. G. Herrera-Soto A. Jensen-Flores J. Bustamante D. B. Onate-Ponce A. (2020). Wnt5a promotes differentiation and development of adult-born neurons in the hippocampus by noncanonical Wnt signaling. *Stem Cells* 38 422–436. 10.1002/stem.3121 31721364

[B7] Artegiani B. Calegari F. (2012). Age-related cognitive decline: Can neural stem cells help us? *Aging* 4 176–186. 10.18632/aging.100446 22466406 PMC3348478

[B8] Bafico A. Liu G. Yaniv A. Gazit A. Aaronson S. A. (2001). Novel mechanism of Wnt signalling inhibition mediated by Dickkopf-1 interaction with LRP6/Arrow. *Nat. Cell Biol.* 3 683–686. 10.1038/35083081 11433302

[B9] Bakker A. Kirwan C. B. Miller M. Stark C. E. (2008). Pattern separation in the human hippocampal CA3 and dentate gyrus. *Science* 319 1640–1642. 10.1126/science.1152882 18356518 PMC2829853

[B10] Ben Abdallah N. M. Slomianka L. Vyssotski A. L. Lipp H. P. (2010). Early age-related changes in adult hippocampal neurogenesis in C57 mice. *Neurobiol. Aging* 31 151–161. 10.1016/j.neurobiolaging.2008.03.002 18455269

[B11] Bengoa-Vergniory N. Kypta R. M. (2015). Canonical and noncanonical Wnt signaling in neural stem/progenitor cells. *Cell. Mol. Life Sci.* 72 4157–4172. 10.1007/s00018-015-2028-6 26306936 PMC11113751

[B12] Bielen H. Houart C. (2014). The Wnt cries many: Wnt regulation of neurogenesis through tissue patterning, proliferation, and asymmetric cell division. *Dev. Neurobiol.* 74 772–780. 10.1002/dneu.22168 24488703

[B13] Bilic J. Huang Y. L. Davidson G. Zimmermann T. Cruciat C. M. Bienz M. (2007). Wnt induces LRP6 signalosomes and promotes dishevelled-dependent LRP6 phosphorylation. *Science* 316 1619–1622. 10.1126/science.1137065 17569865

[B14] Boldrini M. Fulmore C. A. Tartt A. N. Simeon L. R. Pavlova I. Poposka V. (2018). Human hippocampal neurogenesis persists throughout aging. *Cell Stem Cell* 22 589–599.e5. 10.1016/j.stem.2018.03.015 29625071 PMC5957089

[B15] Bonaguidi M. A. Wheeler M. A. Shapiro J. S. Stadel R. P. Sun G. J. Ming G. L. (2011). *In vivo* clonal analysis reveals self-renewing and multipotent adult neural stem cell characteristics. *Cell* 145 1142–1155. 10.1016/j.cell.2011.05.024 21664664 PMC3124562

[B16] Bovolenta P. Rodriguez J. Esteve P. (2006). Frizzled/RYK mediated signalling in axon guidance. *Development* 133 4399–4408. 10.1242/dev.02592 17035295

[B17] Brown J. P. Couillard-Despres S. Cooper-Kuhn C. M. Winkler J. Aigner L. Kuhn H. G. (2003). Transient expression of doublecortin during adult neurogenesis. *J. Comp. Neurol.* 467 1–10. 10.1002/cne.10874 14574675

[B18] Buechling T. Bartscherer K. Ohkawara B. Chaudhary V. Spirohn K. Niehrs C. (2010). Wnt/Frizzled signaling requires dPRR, the Drosophila homolog of the prorenin receptor. *Curr. Biol.* 20 1263–1268. 10.1016/j.cub.2010.05.028 20579883

[B19] Butler M. T. Wallingford J. B. (2017). Planar cell polarity in development and disease. *Nat. Rev. Mol. Cell Biol.* 18 375–388. 10.1038/nrm.2017.11 28293032 PMC5826606

[B20] Caricasole A. Copani A. Caraci F. Aronica E. Rozemuller A. J. Caruso A. (2004). Induction of Dickkopf-1, a negative modulator of the Wnt pathway, is associated with neuronal degeneration in Alzheimer’s brain. *J. Neurosci.* 24 6021–6027. 10.1523/jneurosci.1381-04.2004 15229249 PMC6729239

[B21] Cheung H. Y. Cheung C. S. Kong C. K. (2001). Determination of bioactive diterpenoids from Andrographis paniculata by micellar electrokinetic chromatography. *J. Chromatogr. A* 930 171–176. 10.1016/s0021-9673(01)01160-8 11681575

[B22] Cho C. H. Yoo K. H. Oliveros A. Paulson S. Hussaini S. M. Q. van Deursen J. M. (2019). sFRP3 inhibition improves age-related cellular changes in BubR1 progeroid mice. *Aging Cell* 18:e12899. 10.1111/acel.12899 30609266 PMC6413750

[B23] Choi S. H. Bylykbashi E. Chatila Z. K. Lee S. W. Pulli B. Clemenson G. D. (2018). Combined adult neurogenesis and BDNF mimic exercise effects on cognition in an Alzheimer’s mouse model. *Science* 361:eaan8821. 10.1126/science.aan8821 30190379 PMC6149542

[B24] Choi S. H. Tanzi R. E. (2019). Is Alzheimer’s disease a neurogenesis disorder? *Cell Stem Cell* 25 7–8. 10.1016/j.stem.2019.06.001 31271749

[B25] Cisternas P. Oliva C. A. Torres V. I. Barrera D. P. Inestrosa N. C. (2019). Presymptomatic treatment with andrographolide improves brain metabolic markers and cognitive behavior in a model of early-onset Alzheimer’s disease. *Front. Cell. Neurosci.* 13:295. 10.3389/fncel.2019.00295 31379502 PMC6657419

[B26] Clevers H. Nusse R. (2012). Wnt/beta-catenin signaling and disease. *Cell* 149 1192–1205. 10.1016/j.cell.2012.05.012 22682243

[B27] Cong F. Schweizer L. Varmus H. (2004). Wnt signals across the plasma membrane to activate the beta-catenin pathway by forming oligomers containing its receptors, Frizzled and LRP. *Development* 131 5103–5115. 10.1242/dev.01318 15459103

[B28] Coras R. Siebzehnrubl F. A. Pauli E. Huttner H. B. Njunting M. Kobow K. (2010). Low proliferation and differentiation capacities of adult hippocampal stem cells correlate with memory dysfunction in humans. *Brain* 133 3359–3372. 10.1093/brain/awq215 20719879

[B29] Crews L. Adame A. Patrick C. Delaney A. Pham E. Rockenstein E. (2010). Increased BMP6 levels in the brains of Alzheimer’s disease patients and APP transgenic mice are accompanied by impaired neurogenesis. *J. Neurosci.* 30 12252–12262. 10.1523/jneurosci.1305-10.2010 20844121 PMC2978735

[B30] Cruciat C. M. Niehrs C. (2013). Secreted and transmembrane wnt inhibitors and activators. *Cold Spring Harb. Perspect. Biol.* 5:a015081. 10.1101/cshperspect.a015081 23085770 PMC3578365

[B31] Cui X. P. Xing Y. Chen J. M. Dong S. W. Ying D. J. Yew D. T. (2011). Wnt/beta-catenin is involved in the proliferation of hippocampal neural stem cells induced by hypoxia. *Ir. J. Med. Sci.* 180 387–393. 10.1007/s11845-010-0566-3 20811817

[B32] Danielson N. B. Kaifosh P. Zaremba J. D. Lovett-Barron M. Tsai J. Denny C. A. (2016). Distinct contribution of adult-born hippocampal granule cells to context encoding. *Neuron* 90 101–112. 10.1016/j.neuron.2016.02.019 26971949 PMC4962695

[B33] De A. (2011). Wnt/Ca2+ signaling pathway: a brief overview. *Acta Biochim. Biophys. Sin.* 43 745–756. 10.1093/abbs/gmr079 21903638

[B34] De Ferrari G. V. Avila M. E. Medina M. A. Perez-Palma E. Bustos B. I. Alarcon M. A. (2014). Wnt/beta-catenin signaling in Alzheimer’s disease. *CNS Neurol. Disord. Drug Targets* 13 745–754. 10.2174/1871527312666131223113900 24365184

[B35] De Ferrari G. V. Inestrosa N. C. (2000). Wnt signaling function in Alzheimer’s disease. *Brain Res. Brain Res. Rev.* 33 1–12. 10.1097/00002093-199501002-00001 10967351

[B36] De Ferrari G. V. Papassotiropoulos A. Biechele T. Wavrant De-Vrieze F. Avila M. E. Major M. B. (2007). Common genetic variation within the low-density lipoprotein receptor-related protein 6 and late-onset Alzheimer’s disease. *Proc. Natl. Acad. Sci. U.S.A.* 104 9434–9439. 10.1073/pnas.0603523104 17517621 PMC1890512

[B37] Demars M. Hu Y. S. Gadadhar A. Lazarov O. (2010). Impaired neurogenesis is an early event in the etiology of familial Alzheimer’s disease in transgenic mice. *J. Neurosci. Res.* 88 2103–2117. 10.1002/jnr.22387 20209626 PMC3696038

[B38] Deng W. Aimone J. B. Gage F. H. (2010). New neurons and new memories: How does adult hippocampal neurogenesis affect learning and memory? *Nat. Rev. Neurosci.* 11 339–350. 10.1038/nrn2822 20354534 PMC2886712

[B39] Dennis C. V. Suh L. S. Rodriguez M. L. Kril J. J. Sutherland G. T. (2016). Human adult neurogenesis across the ages: an immunohistochemical study. *Neuropathol. Appl. Neurobiol.* 42 621–638. 10.1111/nan.12337 27424496 PMC5125837

[B40] Denny C. A. Burghardt N. S. Schachter D. M. Hen R. Drew M. R. (2012). 4- to 6-week-old adult-born hippocampal neurons influence novelty-evoked exploration and contextual fear conditioning. *Hippocampus* 22 1188–1201. 10.1002/hipo.20964 21739523 PMC3193906

[B41] Devenport D. (2014). The cell biology of planar cell polarity. *J. Cell Biol.* 207 171–179. 10.1083/jcb.201408039 25349257 PMC4210441

[B42] Donovan M. H. Yazdani U. Norris R. D. Games D. German D. C. Eisch A. J. (2006). Decreased adult hippocampal neurogenesis in the PDAPP mouse model of Alzheimer’s disease. *J. Comp. Neurol.* 495 70–83. 10.1002/cne.20840 16432899

[B43] Drew L. J. Kheirbek M. A. Luna V. M. Denny C. A. Cloidt M. A. Wu M. V. (2016). Activation of local inhibitory circuits in the dentate gyrus by adult-born neurons. *Hippocampus* 26 763–778. 10.1002/hipo.22557 26662922 PMC4867135

[B44] Ekonomou A. Savva G. M. Brayne C. Forster G. Francis P. T. Johnson M. (2015). Stage-specific changes in neurogenic and glial markers in Alzheimer’s disease. *Biol. Psychiatry* 77 711–719. 10.1016/j.biopsych.2014.05.021 25022604

[B45] Encinas J. M. Michurina T. V. Peunova N. Park J. H. Tordo J. Peterson D. A. (2011). Division-coupled astrocytic differentiation and age-related depletion of neural stem cells in the adult hippocampus. *Cell Stem Cell* 8 566–579. 10.1016/j.stem.2011.03.010 21549330 PMC3286186

[B46] Eriksson P. S. Perfilieva E. Bjork-Eriksson T. Alborn A. M. Nordborg C. Peterson D. A. (1998). Neurogenesis in the adult human hippocampus. *Nat. Med.* 4 1313–1317. 9809557 10.1038/3305

[B47] Faigle R. Song H. (2013). Signaling mechanisms regulating adult neural stem cells and neurogenesis. *Biochim. Biophys. Acta* 1830 2435–2448. 10.1016/j.bbagen.2012.09.002 22982587 PMC3541438

[B48] Fiorentini A. Rosi M. C. Grossi C. Luccarini I. Casamenti F. (2010). Lithium improves hippocampal neurogenesis, neuropathology and cognitive functions in APP mutant mice. *PLoS One* 5:e14382. 10.1371/journal.pone.0014382 21187954 PMC3004858

[B49] Folke J. Pakkenberg B. Brudek T. (2019). Impaired Wnt signaling in the prefrontal cortex of Alzheimer’s disease. *Mol. Neurobiol.* 56 873–891. 10.1007/s12035-018-1103-z 29804228

[B50] Freese J. L. Pino D. Pleasure S. J. (2010). Wnt signaling in development and disease. *Neurobiol. Dis.* 38 148–153. 19765659 10.1016/j.nbd.2009.09.003 PMC2854277

[B51] Galan L. Gomez-Pinedo U. Guerrero A. Garcia-Verdugo J. M. Matias-Guiu J. (2017). Amyotrophic lateral sclerosis modifies progenitor neural proliferation in adult classic neurogenic brain niches. *BMC Neurol.* 17:173. 10.1186/s12883-017-0956-5 28874134 PMC5585932

[B52] Gao B. Song H. Bishop K. Elliot G. Garrett L. English M. A. (2011). Wnt signaling gradients establish planar cell polarity by inducing Vangl2 phosphorylation through Ror2. *Dev. Cell* 20 163–176. 10.1016/j.devcel.2011.01.001 21316585 PMC3062198

[B53] Gao Z. Ure K. Ables J. L. Lagace D. C. Nave K. A. Goebbels S. (2009). Neurod1 is essential for the survival and maturation of adult-born neurons. *Nat. Neurosci.* 12 1090–1092. 10.1038/nn.2385 19701197 PMC3365543

[B54] Garbe D. S. Ring R. H. (2012). Investigating tonic Wnt signaling throughout the adult CNS and in the hippocampal neurogenic niche of BatGal and ins-TopGal mice. *Cell. Mol. Neurobiol.* 32 1159–1174. 10.1007/s10571-012-9841-3 22491991 PMC11498517

[B55] Ge S. Goh E. L. Sailor K. A. Kitabatake Y. Ming G. L. Song H. (2006). GABA regulates synaptic integration of newly generated neurons in the adult brain. *Nature* 439 589–593. 10.1038/nature04404 16341203 PMC1420640

[B56] Goncalves J. T. Bloyd C. W. Shtrahman M. Johnston S. T. Schafer S. T. Parylak S. L. (2016). *In vivo* imaging of dendritic pruning in dentate granule cells. *Nat. Neurosci*. 19 788–791. 10.1038/nn.4301 27135217 PMC4941946

[B57] Gordon M. D. Nusse R. (2006). Wnt signaling: multiple pathways, multiple receptors, and multiple transcription factors. *J. Biol. Chem.* 281 22429–22433. 10.1074/jbc.r600015200 16793760

[B58] Gould E. Reeves A. J. Fallah M. Tanapat P. Gross C. G. Fuchs E. (1999). Hippocampal neurogenesis in adult Old World primates. *Proc. Natl. Acad. Sci. U.S.A.* 96 5263–5267. 10.1073/pnas.96.9.5263 10220454 PMC21852

[B59] Green J. Nusse R. van Amerongen R. (2014). The role of Ryk and Ror receptor tyrosine kinases in Wnt signal transduction. *Cold Spring Harb. Perspect. Biol.* 6:a009175. 10.1101/cshperspect.a009175 24370848 PMC3941236

[B60] Grumolato L. Liu G. Mong P. Mudbhary R. Biswas R. Arroyave R. (2010). Canonical and noncanonical Wnts use a common mechanism to activate completely unrelated coreceptors. *Genes Dev.* 24 2517–2530. 10.1101/gad.1957710 21078818 PMC2975928

[B61] Gu Y. Arruda-Carvalho M. Wang J. Janoschka S. R. Josselyn S. A. Frankland P. W. (2012). Optical controlling reveals time-dependent roles for adult-born dentate granule cells. *Nat. Neurosci.* 15 1700–1706. 10.1038/nn.3260 23143513 PMC3509272

[B62] Hamilton L. K. Aumont A. Julien C. Vadnais A. Calon F. Fernandes K. J. (2010). Widespread deficits in adult neurogenesis precede plaque and tangle formation in the 3xTg mouse model of Alzheimer’s disease. *Eur. J. Neurosci.* 32 905–920. 10.1111/j.1460-9568.2010.07379.x 20726889

[B63] Hattiangady B. Shetty A. K. (2008). Aging does not alter the number or phenotype of putative stem/progenitor cells in the neurogenic region of the hippocampus. *Neurobiol. Aging* 29 129–147. 10.1016/j.neurobiolaging.2006.09.015 17092610 PMC3612500

[B64] Heppt J. Wittmann M.-T. Zhang J. Vogt-Weisenhorn D. Prakash N. Wurst W. (2020). Canonical Wnt-signaling modulates the tempo of dendritic growth of adult-born hippocampal neurons. *bioRxiv* [Preprint]. 10.1101/2020.01.14.905919 PMC7604596 32929771

[B65] Hermle T. Guida M. C. Beck S. Helmstadter S. Simons M. (2013). Drosophila ATP6AP2/VhaPRR functions both as a novel planar cell polarity core protein and a regulator of endosomal trafficking. *EMBO J.* 32 245–259. 10.1038/emboj.2012.323 23292348 PMC3553382

[B66] Hollands C. Bartolotti N. Lazarov O. (2016). Alzheimer’s disease and hippocampal adult neurogenesis; exploring shared mechanisms. *Front. Neurosci.* 10:178. 10.3389/fnins.2016.00178 27199641 PMC4853383

[B67] Hsieh J. Nakashima K. Kuwabara T. Mejia E. Gage F. H. (2004). Histone deacetylase inhibition-mediated neuronal differentiation of multipotent adult neural progenitor cells. *Proc. Natl. Acad. Sci. U.S.A.* 101 16659–16664. 10.1073/pnas.0407643101 15537713 PMC527137

[B68] Hsieh J. C. Kodjabachian L. Rebbert M. L. Rattner A. Smallwood P. M. Samos C. H. (1999). A new secreted protein that binds to Wnt proteins and inhibits their activities. *Nature* 398 431–436. 10.1038/18899 10201374

[B69] Hu Y. S. Xu P. Pigino G. Brady S. T. Larson J. Lazarov O. (2010). Complex environment experience rescues impaired neurogenesis, enhances synaptic plasticity, and attenuates neuropathology in familial Alzheimer’s disease-linked APPswe/PS1DeltaE9 mice. *FASEB J.* 24 1667–1681. 10.1096/fj.09-136945 20086049 PMC4050966

[B70] Huang H. C. Klein P. S. (2004). The Frizzled family: receptors for multiple signal transduction pathways. *Genome Biol.* 5:234. 10.1186/gb-2004-5-7-234 15239825 PMC463283

[B71] Humphries C. E. Kohli M. A. Nathanson L. Whitehead P. Beecham G. Martin E. (2015). Integrated whole transcriptome and DNA methylation analysis identifies gene networks specific to late-onset Alzheimer’s disease. *J. Alzheimers Dis.* 44 977–987. 10.3233/JAD-141989 25380588

[B72] Inestrosa N. C. Varela-Nallar L. (2014). Wnt signaling in the nervous system and in Alzheimer’s disease. *J. Mol. Cell Biol.* 6 64–74. 10.1093/jmcb/mjt051 24549157

[B73] Inestrosa N. C. Varela-Nallar L. (2015). Wnt signalling in neuronal differentiation and development. *Cell Tissue Res.* 359 215–223. 10.1007/s00441-014-1996-4 25234280

[B74] Ishitani T. Kishida S. Hyodo-Miura J. Ueno N. Yasuda J. Waterman M. (2003). The TAK1-NLK mitogen-activated protein kinase cascade functions in the Wnt-5a/Ca(2+) pathway to antagonize Wnt/beta-catenin signaling. *Mol. Cell. Biol.* 23 131–139. 10.1128/mcb.23.1.131-139.2003 12482967 PMC140665

[B75] Israsena N. Hu M. Fu W. Kan L. Kessler J. A. (2004). The presence of FGF2 signaling determines whether beta-catenin exerts effects on proliferation or neuronal differentiation of neural stem cells. *Dev. Biol.* 268 220–231. 10.1016/j.ydbio.2003.12.024 15031118

[B76] Jackstadt R. Hodder M. C. Sansom O. J. (2020). WNT and β-catenin in cancer: genes and therapy. *Annu. Rev. Cancer Biol.* 4 177–196.

[B77] Jang M. H. Bonaguidi M. A. Kitabatake Y. Sun J. Song J. Kang E. (2013). Secreted frizzled-related protein 3 regulates activity-dependent adult hippocampal neurogenesis. *Cell Stem Cell* 12 215–223. 10.1016/j.stem.2012.11.021 23395446 PMC3569732

[B78] Jones C. Chen P. (2007). Planar cell polarity signaling in vertebrates. *Bioessays* 29 120–132. 10.1002/bies.20526 17226800 PMC4158832

[B79] Kalamakis G. Brune D. Ravichandran S. Bolz J. Fan W. Ziebell F. (2019). Quiescence modulates stem cell maintenance and regenerative capacity in the aging brain. *Cell* 176 1407–1419.e14. 10.1016/j.cell.2019.01.040 30827680

[B80] Karalay O. Doberauer K. Vadodaria K. C. Knobloch M. Berti L. Miquelajauregui A. (2011). Prospero-related homeobox 1 gene (Prox1) is regulated by canonical Wnt signaling and has a stage-specific role in adult hippocampal neurogenesis. *Proc. Natl. Acad. Sci. U.S.A.* 108 5807–5812. 10.1073/pnas.1013456108 21436036 PMC3078392

[B81] Kase Y. Otsu K. Shimazaki T. Okano H. (2019). Involvement of p38 in age-related decline in adult neurogenesis via modulation of Wnt signaling. *Stem Cell Rep.* 12 1313–1328. 10.1016/j.stemcr.2019.04.010 31080114 PMC6565990

[B82] Kempermann G. Jessberger S. Steiner B. Kronenberg G. (2004). Milestones of neuronal development in the adult hippocampus. *Trends Neurosci.* 27 447–452. 10.1016/j.tins.2004.05.013 15271491

[B83] Knoth R. Singec I. Ditter M. Pantazis G. Capetian P. Meyer R. P. (2010). Murine features of neurogenesis in the human hippocampus across the lifespan from 0 to 100 years. *PLoS One* 5:e8809. 10.1371/journal.pone.0008809 20126454 PMC2813284

[B84] Kohler S. J. Williams N. I. Stanton G. B. Cameron J. L. Greenough W. T. (2011). Maturation time of new granule cells in the dentate gyrus of adult macaque monkeys exceeds six months. *Proc. Natl. Acad. Sci. U.S.A.* 108 10326–10331. 10.1073/pnas.1017099108 21646517 PMC3121825

[B85] Kohn A. D. Moon R. T. (2005). Wnt and calcium signaling: beta-catenin-independent pathways. *Cell Calcium* 38 439–446. 10.1016/j.ceca.2005.06.022 16099039

[B86] Koval A. Katanaev V. L. (2011). Wnt3a stimulation elicits G-protein-coupled receptor properties of mammalian Frizzled proteins. *Biochem. J.* 433 435–440. 10.1042/BJ20101878 21128903

[B87] Kronenberg G. Reuter K. Steiner B. Brandt M. D. Jessberger S. Yamaguchi M. (2003). Subpopulations of proliferating cells of the adult hippocampus respond differently to physiologic neurogenic stimuli. *J. Comp. Neurol.* 467 455–463. 10.1002/cne.10945 14624480

[B88] Kuhl M. Sheldahl L. C. Malbon C. C. Moon R. T. (2000a). Ca(2+)/calmodulin-dependent protein kinase II is stimulated by Wnt and Frizzled homologs and promotes ventral cell fates in Xenopus. *J. Biol. Chem.* 275 12701–12711. 10.1074/jbc.275.17.12701 10777564

[B89] Kuhl M. Sheldahl L. C. Park M. Miller J. R. Moon R. T. (2000b). The Wnt/Ca2+ pathway: a new vertebrate Wnt signaling pathway takes shape. *Trends Genet.* 16 279–283. 10.1016/s0168-9525(00)02028-x 10858654

[B90] Kuhn H. G. Dickinson-Anson H. Gage F. H. (1996). Neurogenesis in the dentate gyrus of the adult rat: age-related decrease of neuronal progenitor proliferation. *J. Neurosci.* 16 2027–2033. 10.1523/JNEUROSCI.16-06-02027.1996 8604047 PMC6578509

[B91] Kuwabara T. Hsieh J. Muotri A. Yeo G. Warashina M. Lie D. C. (2009). Wnt-mediated activation of NeuroD1 and retro-elements during adult neurogenesis. *Nat. Neurosci.* 12 1097–1105. 10.1038/nn.2360 19701198 PMC2764260

[B92] Lacefield C. O. Itskov V. Reardon T. Hen R. Gordon J. A. (2012). Effects of adult-generated granule cells on coordinated network activity in the dentate gyrus. *Hippocampus* 22 106–116. 10.1002/hipo.20860 20882540 PMC3282563

[B93] Lavado A. Lagutin O. V. Chow L. M. Baker S. J. Oliver G. (2010). Prox1 is required for granule cell maturation and intermediate progenitor maintenance during brain neurogenesis. *PLoS Biol.* 8:e1000460. 10.1371/journal.pbio.1000460 20808958 PMC2923090

[B94] Lazarov O. Hollands C. (2016). Hippocampal neurogenesis: Learning to remember. *Prog. Neurobiol.* 138-140 1–18. 10.1016/j.pneurobio.2015.12.006 26855369 PMC4828289

[B95] Leuner B. Kozorovitskiy Y. Gross C. G. Gould E. (2007). Diminished adult neurogenesis in the marmoset brain precedes old age. *Proc. Natl. Acad. Sci. U.S.A.* 104 17169–17173. 10.1073/pnas.0708228104 17940008 PMC2040400

[B96] Li B. Yamamori H. Tatebayashi Y. Shafit-Zagardo B. Tanimukai H. Chen S. (2008). Failure of neuronal maturation in Alzheimer disease dentate gyrus. *J. Neuropathol. Exp. Neurol.* 67 78–84. 10.1097/nen.0b013e318160c5db 18091557 PMC3191920

[B97] Lie D. C. Colamarino S. A. Song H. J. Desire L. Mira H. Consiglio A. (2005). Wnt signalling regulates adult hippocampal neurogenesis. *Nature* 437 1370–1375. 10.1038/nature04108 16251967

[B98] Logan C. Y. Nusse R. (2004). The Wnt signaling pathway in development and disease. *Annu. Rev. Cell Dev. Biol.* 20 781–810. 10.1146/annurev.cellbio.20.010403.113126 15473860

[B99] Lucassen P. J. Stumpel M. W. Wang Q. Aronica E. (2010). Decreased numbers of progenitor cells but no response to antidepressant drugs in the hippocampus of elderly depressed patients. *Neuropharmacology* 58 940–949. 10.1016/j.neuropharm.2010.01.012 20138063

[B100] MacDonald B. T. Tamai K. He X. (2009). Wnt/beta-catenin signaling: components, mechanisms, and diseases. *Dev. Cell* 17 9–26. 10.1016/j.devcel.2009.06.016 19619488 PMC2861485

[B101] Mardones M. D. Andaur G. A. Varas-Godoy M. Henriquez J. F. Salech F. Behrens M. I. (2016). Frizzled-1 receptor regulates adult hippocampal neurogenesis. *Mol. Brain* 9:29. 10.1186/s13041-016-0209-3 26980182 PMC4791773

[B102] Marin-Burgin A. Mongiat L. A. Pardi M. B. Schinder A. F. (2012). Unique processing during a period of high excitation/inhibition balance in adult-born neurons. *Science* 335 1238–1242. 10.1126/science.1214956 22282476 PMC3385415

[B103] Mathews K. J. Allen K. M. Boerrigter D. Ball H. Shannon Weickert C. Double K. L. (2017). Evidence for reduced neurogenesis in the aging human hippocampus despite stable stem cell markers. *Aging Cell* 16 1195–1199. 10.1111/acel.12641 28766905 PMC5595679

[B104] Mentink R. A. Rella L. Radaszkiewicz T. W. Gybel T. Betist M. C. Bryja V. (2018). The planar cell polarity protein VANG-1/Vangl negatively regulates Wnt/beta-catenin signaling through a Dvl dependent mechanism. *PLoS Genet.* 14:e1007840. 10.1371/journal.pgen.1007840 30532125 PMC6307821

[B105] Mikels A. J. Nusse R. (2006). Purified Wnt5a protein activates or inhibits beta-catenin-TCF signaling depending on receptor context. *PLoS Biol.* 4:e115. 10.1371/journal.pbio.0040115 16602827 PMC1420652

[B106] Miranda C. J. Braun L. Jiang Y. Hester M. E. Zhang L. Riolo M. (2012). Aging brain microenvironment decreases hippocampal neurogenesis through Wnt-mediated survivin signaling. *Aging Cell* 11 542–552. 10.1111/j.1474-9726.2012.00816.x 22404871 PMC3350615

[B107] Moreno-Jimenez E. P. Flor-Garcia M. Terreros-Roncal J. Rabano A. Cafini F. Pallas-Bazarra N. (2019). Adult hippocampal neurogenesis is abundant in neurologically healthy subjects and drops sharply in patients with Alzheimer’s disease. *Nat. Med.* 25 554–560. 10.1038/s41591-019-0375-9 30911133

[B108] Nusse R. Varmus H. E. (1982). Many tumors induced by the mouse mammary tumor virus contain a provirus integrated in the same region of the host genome. *Cell* 31 99–109. 10.1016/0092-8674(82)90409-3 6297757

[B109] O’Brien R. J. Wong P. C. (2011). Amyloid precursor protein processing and Alzheimer’s disease. *Annu. Rev. Neurosci.* 34 185–204. 10.1146/annurev-neuro-061010-113613 21456963 PMC3174086

[B110] Okamoto M. Inoue K. Iwamura H. Terashima K. Soya H. Asashima M. (2011). Reduction in paracrine Wnt3 factors during aging causes impaired adult neurogenesis. *FASEB J.* 25 3570–3582. 10.1096/fj.11-184697 21746862

[B111] Olariu A. Cleaver K. M. Cameron H. A. (2007). Decreased neurogenesis in aged rats results from loss of granule cell precursors without lengthening of the cell cycle. *J. Comp. Neurol.* 501 659–667. 10.1002/cne.21268 17278139

[B112] Oliva C. A. Montecinos-Oliva C. Inestrosa N. C. (2018). Wnt signaling in the central nervous system: new insights in health and disease. *Prog. Mol. Biol. Transl. Sci.* 153 81–130. 10.1016/bs.pmbts.2017.11.018 29389523

[B113] Ortiz-Matamoros A. Arias C. (2019). Differential changes in the number and morphology of the new neurons after chronic infusion of Wnt7a, Wnt5a, and Dkk-1 in the adult hippocampus *in vivo*. *Anat. Rec.* 302 1647–1657. 10.1002/ar.24069 30635974

[B114] Panossian A. Hovhannisyan A. Mamikonyan G. Abrahamian H. Hambardzumyan E. Gabrielian E. (2000). Pharmacokinetic and oral bioavailability of andrographolide from Andrographis paniculata fixed combination Kan Jang in rats and human. *Phytomedicine* 7 351–364. 10.1016/S0944-7113(00)80054-9 11081986

[B115] Qu Q. Sun G. Murai K. Ye P. Li W. Asuelime G. (2013). Wnt7a regulates multiple steps of neurogenesis. *Mol. Cell. Biol.* 33 2551–2559. 10.1128/MCB.00325-13 23629626 PMC3700117

[B116] Rattner A. Hsieh J. C. Smallwood P. M. Gilbert D. J. Copeland N. G. Jenkins N. A. (1997). A family of secreted proteins contains homology to the cysteine-rich ligand-binding domain of frizzled receptors. *Proc. Natl. Acad. Sci. U.S.A.* 94 2859–2863. 10.1073/pnas.94.7.2859 9096311 PMC20287

[B117] Richetin K. Leclerc C. Toni N. Gallopin T. Pech S. Roybon L. (2015). Genetic manipulation of adult-born hippocampal neurons rescues memory in a mouse model of Alzheimer’s disease. *Brain* 138 440–455. 10.1093/brain/awu354 25518958

[B118] Riise J. Plath N. Pakkenberg B. Parachikova A. (2015). Aberrant Wnt signaling pathway in medial temporal lobe structures of Alzheimer’s disease. *J. Neural Transm.* 122 1303–1318. 10.1007/s00702-015-1375-7 25680440

[B119] Rios-Esteves J. Haugen B. Resh M. D. (2014). Identification of key residues and regions important for porcupine-mediated Wnt acylation. *J. Biol. Chem.* 289 17009–17019. 10.1074/jbc.M114.561209 24798332 PMC4059143

[B120] Rios-Esteves J. Resh M. D. (2013). Stearoyl CoA desaturase is required to produce active, lipid-modified Wnt proteins. *Cell Rep.* 4 1072–1081. 10.1016/j.celrep.2013.08.027 24055053 PMC3845236

[B121] Rodriguez J. J. Jones V. C. Tabuchi M. Allan S. M. Knight E. M. LaFerla F. M. (2008). Impaired adult neurogenesis in the dentate gyrus of a triple transgenic mouse model of Alzheimer’s disease. *PLoS One* 3:e2935. 10.1371/journal.pone.0002935 18698410 PMC2492828

[B122] Rodriguez J. J. Noristani H. N. Olabarria M. Fletcher J. Somerville T. D. Yeh C. Y. (2011). Voluntary running and environmental enrichment restores impaired hippocampal neurogenesis in a triple transgenic mouse model of Alzheimer’s disease. *Curr. Alzheimer Res.* 8 707–717. 10.2174/156720511797633214 21453244

[B123] Rosi M. C. Luccarini I. Grossi C. Fiorentini A. Spillantini M. G. Prisco A. (2010). Increased Dickkopf-1 expression in transgenic mouse models of neurodegenerative disease. *J. Neurochem.* 112 1539–1551. 10.1111/j.1471-4159.2009.06566.x 20050968

[B124] Roy N. S. Wang S. Jiang L. Kang J. Benraiss A. Harrison-Restelli C. (2000). In vitro neurogenesis by progenitor cells isolated from the adult human hippocampus. *Nat. Med*. 6 271–277. 10.1038/73119 10700228

[B125] Saneyoshi T. Kume S. Amasaki Y. Mikoshiba K. (2002). The Wnt/calcium pathway activates NF-AT and promotes ventral cell fate in Xenopus embryos. *Nature* 417 295–299. 10.1038/417295a 12015605

[B126] Sato A. Yamamoto H. Sakane H. Koyama H. Kikuchi A. (2010). Wnt5a regulates distinct signalling pathways by binding to Frizzled2. *EMBO J.* 29 41–54. 10.1038/emboj.2009.322 19910923 PMC2808370

[B127] Schafer S. T. Han J. Pena M. von Bohlen Und Halbach O. Peters J. Gage F. H. (2015). The Wnt Adaptor Protein ATP6AP2 regulates multiple stages of adult hippocampal neurogenesis. *J. Neurosci.* 35 4983–4998. 10.1523/JNEUROSCI.4130-14.2015 25810528 PMC4389597

[B128] Schulte G. (2010). International Union of Basic and Clinical Pharmacology. LXXX. The class Frizzled receptors. *Pharmacol. Rev.* 62 632–667. 10.1124/pr.110.002931 21079039

[B129] Schwarz T. J. Ebert B. Lie D. C. (2012). Stem cell maintenance in the adult mammalian hippocampus: a matter of signal integration? *Dev. Neurobiol*. 72 1006–1015. 10.1002/dneu.22026 22488809

[B130] Seib D. R. Corsini N. S. Ellwanger K. Plaas C. Mateos A. Pitzer C. (2013). Loss of Dickkopf-1 restores neurogenesis in old age and counteracts cognitive decline. *Cell Stem Cell.* 12 204–214. 10.1016/j.stem.2012.11.010 23395445

[B131] Seib D. R. Martin-Villalba A. (2015). Neurogenesis in the normal ageing hippocampus: a mini-review. *Gerontology* 61 327–335. 10.1159/000368575 25471300

[B132] Selkoe D. J. Hardy J. (2016). The amyloid hypothesis of Alzheimer’s disease at 25 years. *EMBO Mol. Med.* 8 595–608. 10.15252/emmm.201606210 27025652 PMC4888851

[B133] Semenov M. Tamai K. He X. (2005). SOST is a ligand for LRP5/LRP6 and a Wnt signaling inhibitor. *J. Biol. Chem.* 280 26770–26775. 10.1074/jbc.M504308200 15908424

[B134] Serafino A. Giovannini D. Rossi S. Cozzolino M. (2020). Targeting the Wnt/beta-catenin pathway in neurodegenerative diseases: recent approaches and current challenges. *Expert Opin. Drug Discov.* 15 803–822. 10.1080/17460441.2020.1746266 32281421

[B135] Serrano F. G. Tapia-Rojas C. Carvajal F. J. Hancke J. Cerpa W. Inestrosa N. C. (2014). Andrographolide reduces cognitive impairment in young and mature AbetaPPswe/PS-1 mice. *Mol. Neurodegener.* 9:61. 10.1186/1750-1326-9-61 25524173 PMC4414355

[B136] Sheldahl L. C. Park M. Malbon C. C. Moon R. T. (1999). Protein kinase C is differentially stimulated by Wnt and Frizzled homologs in a G-protein-dependent manner. *Curr. Biol.* 9 695–698. 10.1016/S0960-9822(99)80310-8 10395542

[B137] Shruster A. Offen D. (2014). Targeting neurogenesis ameliorates danger assessment in a mouse model of Alzheimer’s disease. *Behav. Brain Res.* 261 193–201. 10.1016/j.bbr.2013.12.028 24388979

[B138] Shtutman M. Zhurinsky J. Simcha I. Albanese C. D’Amico M. Pestell R. (1999). The cyclin D1 gene is a target of the beta-catenin/LEF-1 pathway. *Proc. Natl. Acad. Sci. U.S.A.* 96 5522–5527. 10.1073/pnas.96.10.5522 10318916 PMC21892

[B139] Snyder J. S. Kee N. Wojtowicz J. M. (2001). Effects of adult neurogenesis on synaptic plasticity in the rat dentate gyrus. *J. Neurophysiol.* 85 2423–2431. 10.1152/jn.2001.85.6.2423 11387388

[B140] Sorrells S. F. Paredes M. F. Cebrian-Silla A. Sandoval K. Qi D. Kelley K. W. (2018). Human hippocampal neurogenesis drops sharply in children to undetectable levels in adults. *Nature* 555 377–381. 10.1038/nature25975 29513649 PMC6179355

[B141] Spalding K. L. Bergmann O. Alkass K. Bernard S. Salehpour M. Huttner H. B. (2013). Dynamics of hippocampal neurogenesis in adult humans. *Cell* 153 1219–1227. 10.1016/j.cell.2013.05.002 23746839 PMC4394608

[B142] Suh H. Deng W. Gage F. H. (2009). Signaling in adult neurogenesis. *Annu. Rev. Cell Dev. Biol.* 25 253–275. 10.1146/annurev.cellbio.042308.113256 19575663

[B143] Sun J. Bonaguidi M. A. Jun H. Guo J. U. Sun G. J. Will B. (2015). A septo-temporal molecular gradient of sfrp3 in the dentate gyrus differentially regulates quiescent adult hippocampal neural stem cell activation. *Mol. Brain* 8:52. 10.1186/s13041-015-0143-9 26337530 PMC4559945

[B144] Tapia J. C. Torres V. A. Rodriguez D. A. Leyton L. Quest A. F. (2006). Casein kinase 2 (CK2) increases survivin expression via enhanced beta-catenin-T cell factor/lymphoid enhancer binding factor-dependent transcription. *Proc. Natl. Acad. Sci. U.S.A.* 103 15079–15084. 10.1073/pnas.0606845103 17005722 PMC1622780

[B145] Tapia-Rojas C. Aranguiz F. Varela-Nallar L. Inestrosa N. C. (2016). Voluntary running attenuates memory loss, decreases neuropathological changes and induces neurogenesis in a mouse model of Alzheimer’s disease. *Brain Pathol.* 26 62–74. 10.1111/bpa.12255 25763997 PMC8029165

[B146] Tapia-Rojas C. Schuller A. Lindsay C. B. Ureta R. C. Mejias-Reyes C. Hancke J. (2015). Andrographolide activates the canonical Wnt signalling pathway by a mechanism that implicates the non-ATP competitive inhibition of GSK-3beta: autoregulation of GSK-3beta *in vivo*. *Biochem. J.* 466 415–430. 10.1042/BJ20140207 25423492

[B147] Tetsu O. McCormick F. (1999). Beta-catenin regulates expression of cyclin D1 in colon carcinoma cells. *Nature* 398 422–426. 10.1038/18884 10201372

[B148] Tiwari S. K. Agarwal S. Seth B. Yadav A. Nair S. Bhatnagar P. (2014). Curcumin-loaded nanoparticles potently induce adult neurogenesis and reverse cognitive deficits in Alzheimer’s disease model via canonical Wnt/beta-catenin pathway. *ACS Nano* 8 76–103. 10.1021/nn405077y 24467380

[B149] Tiwari S. K. Seth B. Agarwal S. Yadav A. Karmakar M. Gupta S. K. (2015). Ethosuximide Induces Hippocampal Neurogenesis and Reverses Cognitive Deficits in an Amyloid-beta Toxin-induced Alzheimer Rat Model via the Phosphatidylinositol 3-Kinase (PI3K)/Akt/Wnt/beta-Catenin Pathway. *J. Biol. Chem.* 290 28540–28558. 10.1074/jbc.M115.652586 26420483 PMC4653709

[B150] Tobin M. K. Musaraca K. Disouky A. Shetti A. Bheri A. Honer W. G. (2019). Human hippocampal neurogenesis persists in aged adults and Alzheimer’s disease patients. *Cell Stem Cell.* 24 974–982.e3. 10.1016/j.stem.2019.05.003 31130513 PMC6608595

[B151] Toda T. Gage F. H. (2018). Review: adult neurogenesis contributes to hippocampal plasticity. *Cell Tissue Res.* 373 693–709. 10.1007/s00441-017-2735-4 29185071

[B152] Toda T. Parylak S. L. Linker S. B. Gage F. H. (2019). The role of adult hippocampal neurogenesis in brain health and disease. *Mol. Psychiatry* 24 67–87. 10.1038/s41380-018-0036-2 29679070 PMC6195869

[B153] Toni N. Schinder A. F. (2015). Maturation and functional integration of new granule cells into the adult hippocampus. *Cold Spring Harb. Perspect. Biol.* 8:a018903. 10.1101/cshperspect.a018903 26637288 PMC4691791

[B154] Topol L. Jiang X. Choi H. Garrett-Beal L. Carolan P. J. Yang Y. (2003). Wnt-5a inhibits the canonical Wnt pathway by promoting GSK-3-independent beta-catenin degradation. *J. Cell Biol.* 162 899–908. 10.1083/jcb.200303158 12952940 PMC2172823

[B155] van Amerongen R. Mikels A. Nusse R. (2008). Alternative wnt signaling is initiated by distinct receptors. *Sci. Signal.* 1:re9. 10.1126/scisignal.135re9 18765832

[B156] van Praag H. Kempermann G. Gage F. H. (1999). Running increases cell proliferation and neurogenesis in the adult mouse dentate gyrus. *Nat. Neurosci.* 2 266–270. 10.1038/6368 10195220

[B157] van Praag H. Schinder A. F. Christie B. R. Toni N. Palmer T. D. Gage F. H. (2002). Functional neurogenesis in the adult hippocampus. *Nature* 415 1030–1034. 10.1038/4151030a 11875571 PMC9284568

[B158] van Praag H. Shubert T. Zhao C. Gage F. H. (2005). Exercise enhances learning and hippocampal neurogenesis in aged mice. *J. Neurosci.* 25 8680–8685. 10.1523/JNEUROSCI.1731-05.2005 16177036 PMC1360197

[B159] Varela-Nallar L. Arredondo S. B. Tapia-Rojas C. Hancke J. Inestrosa N. C. (2015). Andrographolide stimulates neurogenesis in the adult hippocampus. *Neural Plast.* 2015:935403. 10.1155/2015/935403 26798521 PMC4700200

[B160] Varela-Nallar L. Inestrosa N. C. (2013). Wnt signaling in the regulation of adult hippocampal neurogenesis. *Front. Cell. Neurosci.* 7:100. 10.3389/fncel.2013.00100 23805076 PMC3693081

[B161] Varela-Nallar L. Rojas-Abalos M. Abbott A. C. Moya E. A. Iturriaga R. Inestrosa N. C. (2014). Chronic hypoxia induces the activation of the Wnt/beta-catenin signaling pathway and stimulates hippocampal neurogenesis in wild-type and APPswe-PS1DeltaE9 transgenic mice *in vivo*. *Front. Cell. Neurosci.* 8:17. 10.3389/fncel.2014.00017 24574965 PMC3918655

[B162] Wexler E. M. Geschwind D. H. Palmer T. D. (2008). Lithium regulates adult hippocampal progenitor development through canonical Wnt pathway activation. *Mol. Psychiatry* 13 285–292. 10.1038/sj.mp.4002093 17968353

[B163] Wexler E. M. Paucer A. Kornblum H. I. Palmer T. D. Geschwind D. H. (2009). Endogenous Wnt signaling maintains neural progenitor cell potency. *Stem Cells* 27 1130–1141. 10.1002/stem.36 19418460 PMC2782960

[B164] Winner B. Winkler J. (2015). Adult neurogenesis in neurodegenerative diseases. *Cold Spring Harb. Perspect. Biol.* 7:a021287. 10.1101/cshperspect.a021287 25833845 PMC4382734

[B165] Yang Y. Mlodzik M. (2015). Wnt-Frizzled/planar cell polarity signaling: cellular orientation by facing the wind (Wnt). *Annu. Rev. Cell Dev. Biol.* 31 623–646. 10.1146/annurev-cellbio-100814-125315 26566118 PMC4673888

[B166] Zeng Q. Long Z. Feng M. Zhao Y. Luo S. Wang K. (2019). Valproic acid stimulates hippocampal neurogenesis via activating the Wnt/beta-catenin signaling pathway in the APP/PS1/Nestin-GFP triple transgenic mouse model of Alzheimer’s disease. *Front. Aging Neurosci.* 11:62. 10.3389/fnagi.2019.00062 30971911 PMC6443965

[B167] Zeng Q. Zheng M. Zhang T. He G. (2016). Hippocampal neurogenesis in the APP/PS1/nestin-GFP triple transgenic mouse model of Alzheimer’s disease. *Neuroscience* 314 64–74. 10.1016/j.neuroscience.2015.11.054 26639620

[B168] Zeng X. Tamai K. Doble B. Li S. Huang H. Habas R. (2005). A dual-kinase mechanism for Wnt co-receptor phosphorylation and activation. *Nature* 438 873–877. 10.1038/nature04185 16341017 PMC2100418

[B169] Zhao C. Teng E. M. Summers R. G. Jr. Ming G. L. Gage F. H. (2006). Distinct morphological stages of dentate granule neuron maturation in the adult mouse hippocampus. *J. Neurosci.* 26 3–11. 10.1523/JNEUROSCI.3648-05.2006 16399667 PMC6674324

[B170] Zheng R. Zhang Z. H. Chen C. Chen Y. Jia S. Z. Liu Q. (2017). Selenomethionine promoted hippocampal neurogenesis via the PI3K-Akt-GSK3β-Wnt pathway in a mouse model of Alzheimer’s disease. *Biochem. Biophys. Res. Commun.* 485 6–15. 10.1016/j.bbrc.2017.01.069 28109879

[B171] Zhu L. Chi T. Zhao X. Yang L. Song S. Lu Q. (2018). Xanthoceraside modulates neurogenesis to ameliorate cognitive impairment in APP/PS1 transgenic mice. *J. Physiol. Sci.* 68 555–565. 10.1007/s12576-017-0561-9 28744803 PMC10717762

